# Carbohydrate antigen 125 in congestive heart failure: ready for clinical application?

**DOI:** 10.3389/fonc.2023.1161723

**Published:** 2023-10-31

**Authors:** Rui Feng, Zhenlu Zhang, Qingkun Fan

**Affiliations:** ^1^ Department of Laboratory Medicine, Wuhan Asian Heart Hospital Affiliated to Wuhan University of Science and Technology, Wuhan, China; ^2^ School of Medicine, Wuhan University of Science and Technology, Wuhan, China

**Keywords:** carbohydrate antigen 125, congestive heart failure, heart failure, N-terminal Pro-B-type natriuretic peptide, tissue congestion

## Abstract

Congestion is the permanent mechanism driving disease progression in patients with acute heart failure (AHF) and also is an important treatment target. However, distinguishing between the two different phenotypes (intravascular congestion and tissue congestion) for personalized treatment remains challenging. Historically, carbohydrate antigen 125 (CA125) has been a frequently used biomarker for the screening, diagnosis, and prognosis of ovarian cancer. Interestingly, CA125 is highly sensitive to tissue congestion and shows potential for clinical monitoring and optimal treatment of congestive heart failure (HF). Furthermore, in terms of right heart function parameters, CA125 levels are more advantageous than other biomarkers of HF. CA125 is expected to become a new biological alternative marker for congestive HF and thereby is expected be widely used in clinical practice.

## 1 Introduction

**Figure 1:**
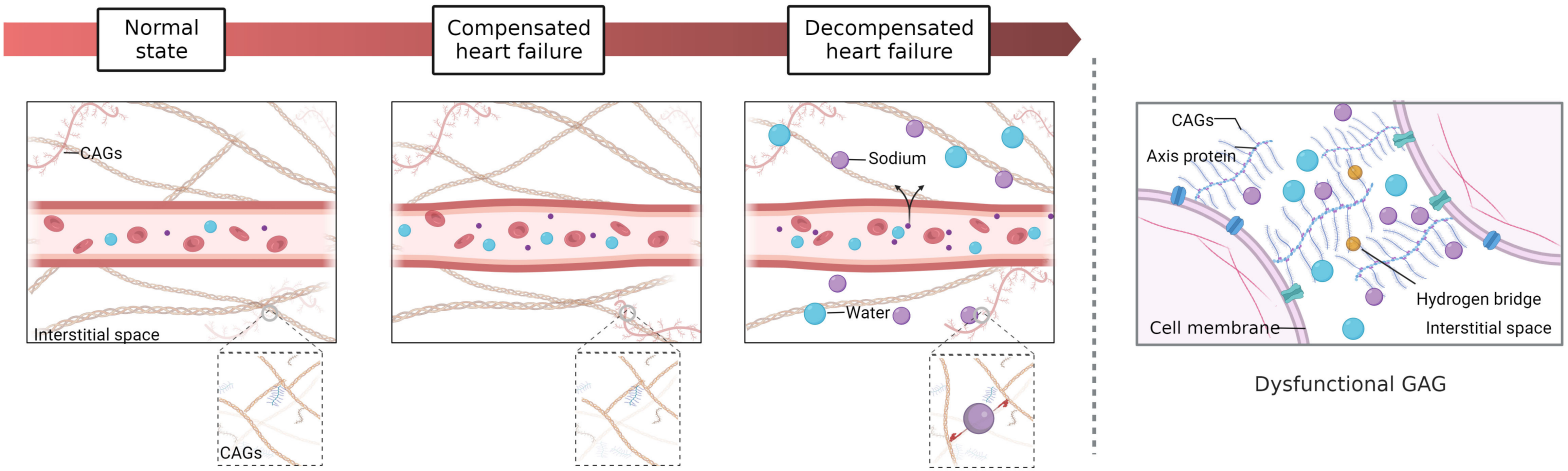
Tissue versus intravascular congestion. Interstitial fluid is formed by filtration through capillaries and subsequently drained by many lymphatic vessels. 1) Normal state: the hydrostatic pressure and osmotic pressure of capillaries are balanced. 2) Compensated HF (isolated circulatory congestion): with the increases in blood volume in capillaries, hydrostatic pressure rises and osmotic pressure decreases, but a balance is generally maintained by the compensatory mechanism. 3) Decompensated HF (circulatory congestion with tissue congestion): During the early stages of decompensated HF, there may be circulatory congestion with subclinical tissue congestion. At this point, apart from weight gain, there may not be any obvious symptoms or signs of congestion. As the condition progresses, there may be an increase in plasma volume and interstitial water, leading to circulatory congestion with tissue congestion and with development of symptoms during exercise and possibly interstitial lung edema (B lines). In the terminal stage, severe dyspnea at rest may even occur. Lymphatic drainage becomes less efficient, and the hydrostatic pressure and osmotic pressure of capillaries continue to increase so that many sodium and water molecules begin to accumulate in the interstitial space. The interstitial space contains a large number of glycosaminoglycans (GAGs), which form a strong network connected by hydrogen bonds and have important regulatory functions. However, high concentrations of sodium eventually alter the conformation of macromolecules in the GAG network, resulting in a loss of interstitial network integrity and buffering capacity. Hence, a slight increase in capillary blood pressure may cause interstitial fluid accumulation during this stage.

According to the 2021 European Society of Cardiology (ESC) Guidelines, the underlying cause of cardiac insufficiency is important in the diagnosis of heart failure (HF). This is because understanding the specific pathophysiology can help determine appropriate treatment options (1). Congestion is the culprit in the progression of HF and end-stage organ damage, which can eventually lead to direct cytotoxicity, myocardial fibrosis, arrhythmia, and pump failure. Congestion symptoms mainly manifest at the pulmonary, systemic or mixed levels. With the increase in venous pressure, plasma gradually infiltrates into the interstitial space. The current research mainly focuses on two different types of congestion, including intravascular congestion and tissue congestion. The majority of patients with congestive HF frequently experience intravascular and tissue congestion (**Figure 1**) (2, 3). However, with the development of precision medicine, it cannot be denied that it is crucial to identify the main phenotype in the formulation of personalized treatment and the assessment of prognosis.

Classical theory suggests that N-terminal pro-B-type natriuretic peptide (NT-proBNP) is the gold standard for the diagnosis of intravascular congestion in patients with congestive HF. Nevertheless, existing studies indicate that NT-proBNP is not an appropriate marker in the assessment of tissue congestion (4–6). Since Nägele et al. (5) first observed in 1999 that the increase in serum carbohydrate antigen 125 (CA125) levels in HF patients was significantly correlated with clinical severity and filling pressure. Numerous studies have been carried out to explore the correlation between CA125 and HF clinical, neurohumoral and hemodynamic data (**Tables 1**, **2**). Therefore, further studies are needed to identify a more comprehensive biomarker to guide treatment and inform prognosis to meet clinical needs. This article aims to provide a better understanding of the contributory role of CA125 in clinical application and shed light on a novel therapeutic target for congestive HF.

**Table 1:** Relationship between CA125 levels and both sides of the heart.

Author	Year	N	Study type	Population	CA125 values	Results
D**’**Aloia et al. (7)	2003	286	Observational	Congestive HF	68 ± 83 U/mL (Range 3–537)	CA125 was strongly negatively correlated with the DT but strongly positively correlated with PAWP and RAP.
Kouris et al. (8)	2005	77	Observational	Congestive HF	22.4 U/mL (11.5-48.9)	CA125 levels were weakly correlated with RVSP and not correlated with LVEF, LVEDD and DT.
Varol et al. (9)	2007	32	Observational	HCM	14.6 ± 23.8 U/mL	There was no significant correlation between the levels of CA125 and E/A ratio, DT, LVMI, LVEDD and blood pressure.
Duman et al. (10)	2008	49	Observational	AHF (NYHA class III- IV)	44.0 U/mL (17.7–140)	Higher levels of serum CA125 were related to NYHA class, serum BNP levels, LAVI and E/e′ increase. In multivariate analysis, serum CA125 levels were significantly correlated with BNP and LAVI.
Vizzardi et al. (11)	2008	200	Observational	Chronic HF	24.79 ± 113.3 U/mL	CA125 levels were weakly positively correlated with E, E/A ratio, MPI and moderately negatively correlated with DT, IVRT.
Yilmaz et al. (12)	2011	150	Retrospectively	Chronic HF	Not Reported	CA125 levels were weakly negatively correlated with EF and weakly positively correlated with PASP. The presences of depressed EF, right ventricular dilatation, and pericardial effusion were identified as independent predictors of high CA125 levels.
Yilmaz et al. (13)	2011	40	Observational	COPD	33.94 U/mL (5.51–351)	CA125 levels were moderately positively correlated with PASP but moderately negatively correlated with TAPSE and tricuspid lateral annulus S velocity.
Karaca et al. (14)	2012	77	Observational	NIDCM	13.3 U/mL (3.8–465.5)	Increased CA125 levels were associated with LV volumes, LVEF, LV filling pressures, PASP, and the degree of functional mitral regurgitation.
Durak et al. (15)	2013	76	Observational	Heart disease and rheumatism	71.05 U/mL (30.70-141.47)	Levels of serum CA125 were weakly positively correlated with serum level of BNP, but not with LVEF and left atrium diameter.
Yilmaz et al. (16)	2014	110	Observational	End-stage renal disease on maintenance hemodialysis	38.78 ± 35.48 U/mL	Serum CA125 levels were moderately positively correlated with pro-BNP and LVEDD, strongly positively correlated with LVESD, and weakly positively correlated with LVMI. Serum CA125 levels were strongly negatively correlated with EF. In contrast, no correlation was found between CA125 levels and diastolic function indices.

CA125, carbohydrate antigen 125; LV, left ventricular; HF, heart failure; DT, deceleration time; PAWP, pulmonary artery wedge pressure; RAP, right atrial pressure; RVSP, right ventricular systolic pressure; LVEF, left ventricular ejection fraction; LVEDD, left ventricular end-diastolic diameter; HCM, hypertrophic cardiomyopathy; LVMI, left ventricular mass index; AHF, acute heart failure; NYHA, New York Heart Association; BNP, brain natriuretic peptide; LAVI, left atrial volume indexed; E/e′: relationship between the E wave and e**’** velocity; E, mitral flow E wave velocity; IVRT, isovolumetric relaxation time; EF, ejection fraction; PASP, pulmonary artery systolic pressure; COPD, chronic obstructive pulmonary disease; TAPSE, tricuspid annular plane systolic excursion; NIDCM, non-ischemic dilated cardiomyopathy; Pro-BNP, Pro-Brain Natriuretic Peptide; LVESD, left ventricular end-systolic diameter.

**Table 2:** Relationship between CA125 and the severity of symptoms, physical fitness and decompensation of heart failure.

Author	Year	N	Study type	Population	CA125 values	Results
Nägele et al. (5)	1999	71	Observational	Chronic HF indication for HTx	401 ± 259 U/mL	CA125 correlated significantly with neurohormones and high-filling pressures.
D**’**Aloia et al. (7)	2003	286	Observational	Congestive HF	68 ± 83 U/mL (range 3–537)	The serum CA125 level showed a correlation with the clinical status and both invasive and non-invasive hemodynamic abnormalities, particularly with the NYHA class, RAP, and PAWP.
Turk et al. (17)	2003	36	Observational	Chronic HF	Patients with pleural effusion: 100.0 ± 129.4 U/mL; Without pleural effusion: 36.5 ± 35.2 U/mL	Serum CA125 levels were higher in patients with HF and pleural effusion compared with both patients without pleural effusion and the control group.
Duman et al. (18)	2003	90	Observational	HF	Not Reported	Elevated CA125 levels were detected in patients with severe symptomatic mitral stenosis and normal LVEF and LV dimensions.
Kouris et al. (8)	2005	77	Observational	Chronic congestive HF	22.4 U/mL (11.5–48.9)	The level of serum CA125 was related to the clinical status (NYHA) and the presence of fluid congestion (pulmonary congestion, ankle edema).
Faggiano et al. (19)	2005	191	Observational	Chronic HF	100 ± 109 U/mL	CA125 levels were linked to both the existence and severity of congestive HF, and that they underwent significant changes in response to medical treatment.
Varol et al. (20)	2005	44	Observational	Chronic HF	81.9 ± 91U/mL	CA125 levels showed significant correlation with the presence and severity of HF, as well as the presence of pleural effusion.
Vizzardi et al. (11)	2008	200	Observational	Chronic HF	24.79 ± 113.3 U/mL	CA125 levels correlated with Doppler mitral flow E/A ratio, isovolumetric relaxation time, and myocardial performance index.
Duman et al. (10)	2008	49	Observational	Advanced HF	44.0 U/mL (17.7-140)	CA125 was significantly correlated with increased LAVI in parallel to increased neurohormonal activation in patients with advanced HF.
Yilmaz et al. (13)	2011	40	Observational	COPD patients hospitalized with exacerbation	33.94 U/mL (5.51–351)	CA125 levels were significantly higher in patients with RV failure, and CA125 levels were finely correlated with markers of RV dysfunction.
Yilmaz et al. (12)	2011	150	Observational	Chronic HF	Not Reported	CA125 was associated with LVEF, RV dilatation, and presence of pericardial effusion.
Karaca et al. (14)	2012	77	Observational	NIDCM	13.3 U/mL (3.8–465.5)	Increased CA125 levels were associated with LV volumes, LVEF, LV filling pressures, PASP, and the degree of functional mitral regurgitation.
Ordu et al. (21)	2012	102	Observational	Chronic HF	Without adverse outcome: 32 ± 59 U/mL; With adverse outcome: 73 ± 74 U/mL	CA125 level significantly correlated with NYHA class, PAP, and NT-proBNP levels.
Huang et al. (22)	2013	191	Observational	AHF	Cut point: 35 U/mL	CA125 levels were associated with the presence of SCE. In the absence of SCE, CA125 levels were also higher in HF patients than in non-HF patients and correlated with systemic inflammation and oxidative stress.
Zhuang et al. (23)	2014	4159 (23 studies)	Meta-analysis	Acute and chronic HF	Not Reported	Positive correlation with natriuretic peptides, inflammatory cytokines, clinical systemic congestion surrogates, and NYHA functional class.
Núñez et al. (24)	2020	516	Observational	Worsening HF	38.6 U/mL (16–125)	Circulating levels of CA125 were associated with a congestion clinical score.
Miñana et al. (25)	2020	2949	Observational	AHF	58.1 U/mL (25–129)	Clinical surrogates of congestion and TR severity were the main factors associated with CA125.
Llàcer et al. (26)	2021	191	Observational	AHF	58 U/mL (22.7–129)	CA125 was independently associated with congestion parameters (inferior vena cava diameter, peripheral edema, and pleural effusion).

CA125, carbohydrate antigen 125; HF, heart failure; AHF, acute heart failure; HTx, heart transplantation; NYHA, New York Heart Association; RAP, right atrial pressure; PAWP, pulmonary artery wedge pressure; LAVI, left atrial volume indexed; COPD, chronic obstructive pulmonary disease; RV, right ventricular; LVEF, left ventricular ejection fraction; NIDCM, non-ischemic dilated cardiomyopathy; PASP, pulmonary artery systolic pressure; LV, left ventricular; PAP, pulmonary artery pressure; NT-proBNP, N-terminal pro-B-type natriuretic peptide; SCE, serous cavity effusion; TR, tricuspid regurgitation.

## 2 Overview of the characteristics of CA125 and congestive heart failure

**Figure 2:**
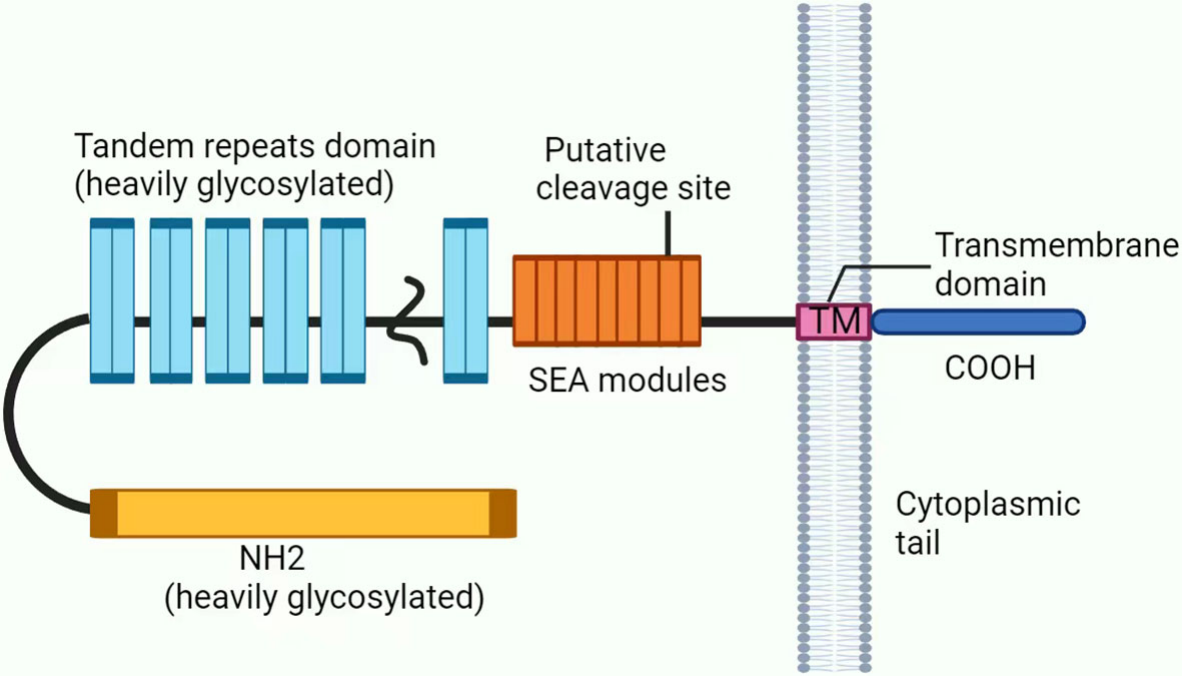
Structure of CA125/MUC16. CA125 is mainly composed of three parts: (1) The N-terminal domain (heavily glycosylated). (2) The tandem repeat domain, which is highly O-glycosylated, has repeating sequences that are high in serine, threonine and proline. (3) The C-terminal domain contains multiple extracellular SEA (sea urchin sperm protein, enterokinase, and agrin) modules, a transmembrane (TM) domain, and a short 31-amino acid cytoplasmic tail (CT). In addition, CA125 is thought to be putatively cleaved at a potential tyrosine phosphorylation site in SEA.

CA125 (also known as MUC16) is a transmembrane protein that is highly glycosylated and has a high molecular weight. It is mainly composed of three parts: the N-terminal domain, tandem repeat domain, and C-terminal domain (**Figure 2**) (27–29). Research shows that CA125 is not expressed in tumor cells but instead originates from the cell surfaces of various tissues of the coelomic epithelium (30). Its main function is to hydrate, lubricate and protect the surface of the epithelial cavity from physical pressure (24, 31, 32). CA125 is considered a valuable biomarker for diagnosing ovarian cancer and evaluating the therapeutic prognosis of patients (31–33).

The exact biological mechanism of CA125 remains unclear, but it is believed that several biological pathways may be involved. (a) It induces the movement of tumor cells through the interaction between cadherin and epithelial-mesenchymal and combines with mesothelin to facilitate the invasion of cells to the peritoneum (34). (b) CA125 not only promotes the progression and metastasis of ovarian cancer but also acts as a barrier for trophoblasts to adhere to the endometrium, protects cancer cells from natural killer (NK) cell-mediated destruction, and controls red blood cell aggregation. (c) CA125 can also act as a barrier for bacterial and viral infections in the ocular epithelium (35, 36). Felder et al. (6) reported that the N-glycan structure presented in CA125 may have a role in regulating both innate and adaptive immune responses. Furthermore, CA125 can function as a lectin anti-receptor and has a strong affinity for galectin-1 and galectin-3 (37). Galectin-3 may also contribute to cardiac remodelling by regulating galectin activity and modifying the quality and hardness of the intercellular matrix.

In addition to malignant tumors, elevated CA125 levels can also be observed in various physiological or pathological conditions such as early pregnancy, menstruation, peritoneal injury and ascites of any cause (34, 38, 39), such as ascites due to cirrhosis (40). Subsequent studies have highlighted congestive HF as a special cause of increased serum CA125 levels. Moreover, CA125 levels are closely associated with the severity of congestion and are often accompanied by significant volume overload and fluid accumulation (41). Indeed, up to two-thirds of patients with AHF exhibit CA125 levels (35-200 U/mL) above the normal range (32, 42), and in patients with stable HF is mostly lower than 35 U/mL. In contrast, patients with ovarian cancer may exhibit serum concentrations as high as 2000-3000 U/mL (43, 44).

**Figure 3:**
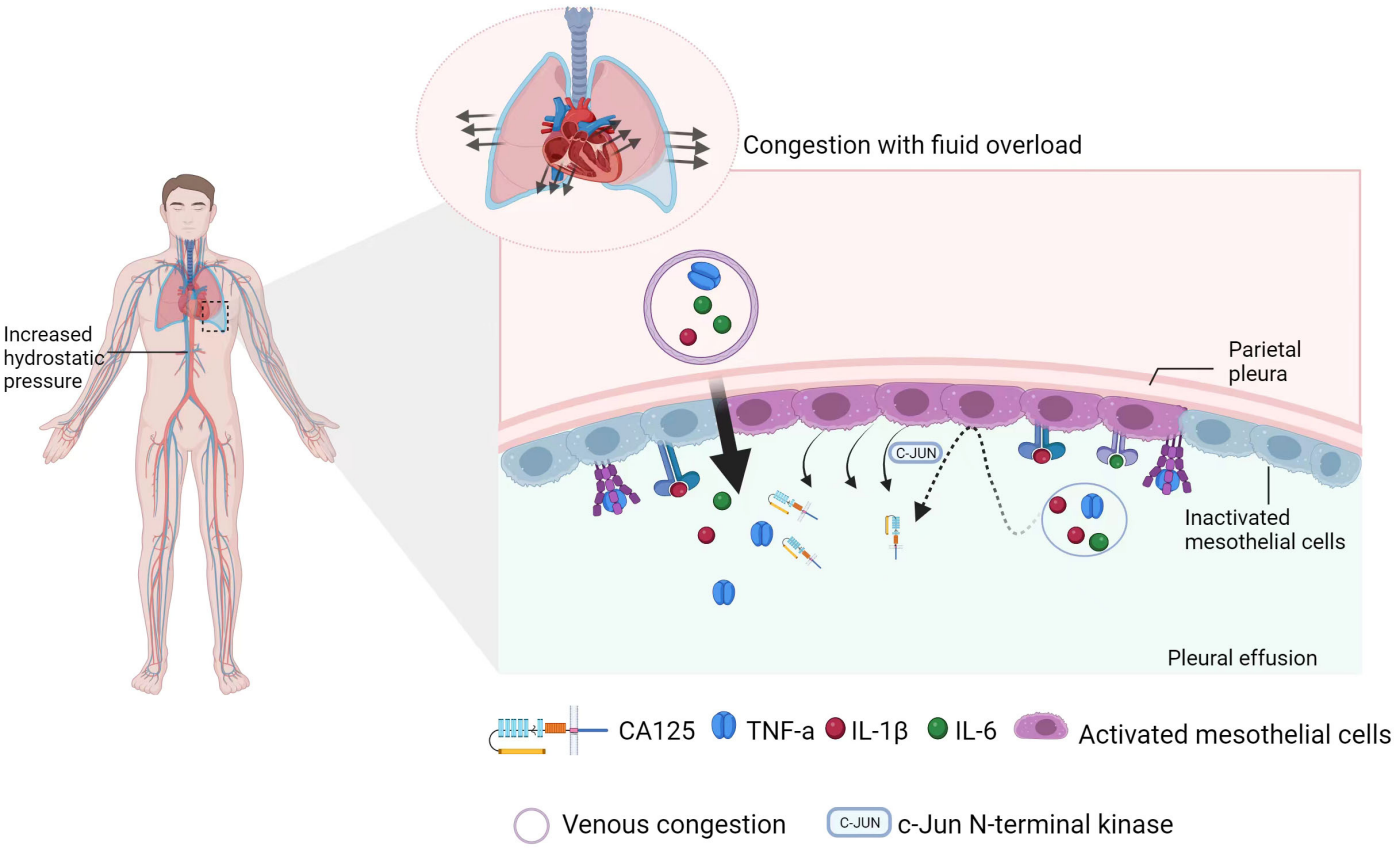
Schematic diagram of CA125 synthesis. On the one hand, intravascular congestion (venous congestion) can lead to endothelial activation, upregulation of inflammatory cytokines, liver dysfunction, and intestinal villus ischemia, which can promote the release of inflammatory factors. However, tissue congestion (pleural effusion) activates mesothelial cells in the pleura through mechanical stress and inflammatory stimuli. This activation initiates the synthesis of CA125 via the JNK pathway. At the same time, changes in cell morphology and membrane stability cause shedding of the extracellular domain of CA125 from mesothelial cells.

The mechanism behind the upregulation of serum CA125 in patients with congestive HF has not been confirmed. In the existing studies, mechanical stress and inflammatory stimulation are considered crucial factors in this process, and two hypotheses have been proposed (**Figure 3**): (1) Mesothelial cells are stimulated by the mechanical stress caused by tissue tension due to fluid overload, leading to the release of CA125 (45, 46). (2) The overexpression of CA125 in mesothelial cells is stimulated by activation of the inflammatory cytokine network (42). According to Colombo et al. (47, 48), venous congestion leads to endothelial activation, upregulation of inflammatory cytokines, hepatic dysfunction, and intestinal villus ischemia. Intestinal villus ischemia can eventually cause abnormal function and loss of barrier function in intestinal epithelial cells, allowing the lipopolysaccharides and endotoxins produced by gram-negative bacteria in the intestinal lumen to enter the circulation. It further aggravates the inflammatory environment that is already established by venous congestion and neurohormonal activity. Fluid overload and inflammatory processes interact to form a vicious cycle in congestive HF. Moreover, mechanical stress and inflammatory stimuli activate the JNK pathway within the cytoplasm of mesothelial cells and jointly initiate the synthesis of CA125 (49–51). There are data showing that interleukin-6 (IL-6), interleukin-10 (IL-10), and tumor necrosis factor-α (TNF-α) can promote CA125 synthesis by stimulating mesothelial cells in the case of HF (46, 48, 52, 53). In an *in vitro* model, Zeillemaker et al. demonstrated that the ability of mesothelial cells to synthesize CA125 is enhanced when they are stimulated (27, 54). It has been suggested that CA125 may act as a secondary cytokine, and its level could be increased due to the activation of primary cytokine networks such as TNF-α and IL-1, 4 (55). However, the relationship between the primary and secondary factors is not fully understood and requires further research. Myocardial remodelling can result in pathological cardiomyocyte hypertrophy and re-expression of embryonic genes. This process is accompanied by the activation of proto-oncogenes, which further stimulate growth factors in the embryo, leading to increased levels of CA125 (56).

It is crucial to provide a comprehensive explanation for elevated CA125 levels to differentiate between ovarian cancer and HF accurately. While fluid retention can cause tissue damage and elevate CA125 levels in both ovarian cancer and HF patients, the level of CA125 is considerably higher in ovarian cancer patients compared to HF patients (57). Furthermore, it is important to note that CA125 alone is not an ideal diagnostic tool for either condition due to its limited specificity and sensitivity (57). Therefore, for better clinical effectiveness in screening and early detection, it should be combined with symptoms/signs, other biomarkers or ultrasound and other multimodal methods.

## 3 Potential clinical applications of CA125

### 3.1 Diagnostic utility of CA125 for heart failure and its LV phenotype

In cases of myocardial hypertrophy and failure, compensatory mechanisms, which mainly include the renin-angiotensin-aldosterone system (RAAS), vasopressin activation, and sympathetic nerve excitation, can cause water and sodium retention, as well as peripheral vasoconstriction. These mechanisms increase the cardiac pre- and postloads, leading to an increase in venous pressure. Additionally, an imbalance of capillary hydrostatic pressure and colloid osmotic pressure in the mesenchyme can result in peripheral edema, serosal cavity effusion, and an increase in the pulmonary capillary wedge pressure (2). B-type natriuretic peptide (BNP) is a neurohormone synthesized in ventricular myocardium that is released into the circulation during ventricular dilatation and pressure overload. As a result, increased circulating natriuretic peptides (NPs) may indicate intravascular and intracardiac congestion rather than tissue congestion. Symptoms and signs, such as ascites, rales, and peripheral edema, are established indicators of tissue congestion, but they are poorly sensitive to the diagnosis of interstitial edema in HF.

In chronic HF patients, those with serous cavity effusion (SCE) had significantly higher serum CA125 levels than those without SCE (22). ROC curve analyses of CA125 and NT-proBNP showed that NT-proBNP was more suitable for predicting the presence of chronic HF than CA125. However, CA125 was found to be a better predictor of chronic HF patients with SCE than NT-proBNP. Additionally, CA125 was identified as an important predictor of AHF with pleural effusion and peripheral edema (21, 32). The increase in CA125 is influenced by the mechanical extension of mesothelial cells from SCE (45, 46). It is not yet clear whether the serum CA125 threshold is still less than 35 U/mL in HF. In HF patients with aortic stenosis (AS), the area under the ROC curve was 0.85 for CA125 and 0.78 for BNP. The best cut-offs were determined to be 10.3 U/mL and 254.64 pg/mL, respectively (58). In patients with chronic obstructive pulmonary disease (COPD), CA125 levels can predict the presence of right ventricular failure with an AUC of 0.902. High levels of CA125 (≥35 U/mL) increase the risk of having a diagnosis of right ventricular failure by echocardiography by 51-fold (13). It is worth noting that during the process of diagnosing HF, several confounding factors need to be ruled out. These include malignant tumors, active inflammatory diseases, and women who are menstruating or pregnant, as these conditions may lead to conflicting results (34, 38, 39). In addition, it is necessary to adjust for the interference of age, sex, obesity, and atrial fibrillation (AF) on the level of CA125 (13, 59, 60). Therefore, a comprehensive evaluation should be conducted before making any clinical explanations.

The relationship between CA125 levels and echocardiographic parameters has produced conflicting results in some studies, particularly in regards to left ventricular function (**Table 1**). In a study of 77 patients with congestive HF, CA125 levels were weakly correlated with right ventricular systolic pressure (RVSP) and were not correlated with Doppler echocardiography E wave deceleration time(DT), left ventricular ejection fraction (LVEF), or left ventricular end diastolic diameter (LVEDD) (8). Duman et al. (8, 10, 18) analyzed the relationship between serum CA125 levels and left ventricular (LV) filling pressure parameters in patients with advanced HF. They also measured plasma BNP and used the left atrial volume index (LAVI) to reflect the severity and duration of LV diastolic dysfunction. The study found a positive correlation between ln CA125 and ln BNP, LAVI, and the ratio of mitral inflow early diastolic velocity to annulus velocity (E/e). However, there was no correlation with ejection fraction (EF), mitral deceleration time (DT), isovolumic relaxation time (IVRT), mitral E-wave velocity, or the ratio of mitral inflow early to late (A) diastolic velocity (E/A). In a study by Varol et al. (9), CA125 was not found to be associated with left ventricular functional parameters. However, Yilmaz et al. (12) discovered that CA125 levels were positively correlated with pulmonary systolic pressure and negatively correlated with ejection fraction. Furthermore, a decrease in EF, right ventricular dilatation, and pericardial effusion were independent predictors of high CA125 levels. Although the relationship between CA125 and left ventricular function parameters is controversial in different studies, the level of CA125 is always related to pulmonary artery wedge pressure and right atrial pressure. This suggests that CA125 may be advantageous in evaluating right ventricular function.

### 3.2 CA125: symptom severity, physical capacity and decompensation

As a biomarker reflecting congestion, CA125 is related to disease severity, hemodynamics and echocardiographic parameters. Patients with HF had a much higher serum level of CA125 than healthy controls (**Table 2**). In a study of 529 patients with AHF, the average plasma level of CA125 was 7-fold higher than that of a control group of asymptomatic HF patients matched for age, sex and cardiovascular risk factor (60). Durak et al. (15) found that the median serum CA125 level was significantly higher in patients with decompensated HF than in those with compensated HF. Kouris et al. (8) used the New York Heart Association (NYHA) classification method and found that the serum CA125 values were higher in patients with NYHA Class III/IV than in those with NYHA Class I/II. Meanwhile, 44 patients with HF were prospectively evaluated, whose CA125 levels were positively correlated with NYHA functional class (61), and the subsequent findings were also consistent with this finding (15). In addition, CA125 levels are also associated with right ventricular enlargement and dysfunction. For example, Nägele et al. (5) found that CA125 values were positively correlated with right atrial pressure and pulmonary capillary wedge pressure in patients with advanced HF receiving heart transplantation. In patients with COPD, CA125 levels were negatively correlated with tricuspid annular plane systolic excursion and tricuspid lateral annulus S velocity but positively correlated with the severity of tricuspid regurgitation (TR) and right atrial size (13). Therefore, CA125 can serve as a useful surrogate index for assessing the severity of HF.

### 3.3 CA125 and prognosis

CA125 has been shown to be associated with poor outcomes in patients with AHF (**Table 3**). A study found that repeated measurements of serum CA125 (3402 observations) over the long term can independently predict the risk of long-term death in a group of 946 patients who were discharged from AHF. Patients with normal serum CA125 levels (median of 31 days) at their first outpatient visit had the lowest risk of death, while those with reduced but not normal levels were at intermediate risk. The changes in serum CA125 levels can be used to predict the long-term prognosis of patients with HF and improve risk stratification (69). The current study found that CA125 was significantly associated with the risk of death and readmission in multiple AHF regimens (7, 32, 42, 69). The BioStat-CHF cohort study found a strong association between CA125 levels and a combined risk of 1-year all-cause death, HF all-cause death, and hospitalization (24). A study of 1111 cases of AHF found that a higher level of CA125 (> 60 U/mL) was associated with increased mortality at 6 months. Another study by Hung et al. (64) reported that adding CA125 to NT-proBNP improved the prediction of hospitalization for female HF patients with preserved ejection fraction (HFpEF). In conclusion, CA125 is a useful short- and long-term prognostic factor for HF patients. When paired with other biomarkers, it can enhance the precision of predicting adverse events.

**Table 3:** Prognostic role of CA125 in patients with heart failure.

Author	Year	Study type	N	Population	Values	Follow-up	Results
D’Aloia et al. (7)	2003	Observational, prospective	286	Congestive HF	Cut point: 35 U/mL	6 ± 3 months	Patients with higher CA125 levels (>35 U/mL)were more likely to die or to be admitted with HF at 6-month follow-up.
Núñez et al. (60)	2007	Observational, prospective	529	AHF	Cut point: 35 U/mL	6 months	Patients with higher CA125 levels(>35 U/mL) were more likely to die within a 6-month follow-up.
Núñez et al. (41)	2010	Observational, prospective	1111	AHF	Cut point: 60 U/mL	6 months	CA125 added prognostic value beyond the information provided by BNP. Their combination enables better 6-month risk stratification.
Mansour et al. (62)	2010	Observational, prospective	172	Acute decompensated HF (African American)	Cut point: 35 U/mL	40-month all-cause mortality, 18-month HF rehospitalization	CA125(>35 U/mL) was found to predict all-cause mortality within 40 months, and the level of CA125 was not related to readmission in 18 months with HF.
Monteiro et al. (63)	2010	Observational, prospective	88	HTx	Cut point: 38 U/mL	13± 7 months after CA125 determination.	CA125 and sodium levels were the only independent predictors of the combined endpoint (death or HTx).
Yilmaz et al. (12)	2011	Observational, retrospective	150	Chronic HF	Cut point: 35 U/mL	8 months (minimum 2, maximum 42 months)	Patients with higher levels of CA125 (>35 u/mL) are more likely to die or be admitted to hospital due to HF.
Hung et al. (64)	2012	Observational, retrospective	158	Women with chronic HF	Cut point: 17.29 U/mL	828.1 days (interquartile range 38 to 1,504.5)	The hospitalization rate of HF patients with CA125 levels(>17.29 U/mL) was the highest. Serum CA125 might be a new biomarker for predicting HFpEF and HF hospitalization in women.
Ordu et al. (21)	2012	Observational, prospective	102	Chronic HF	Cut point: 32 U/mL	14 ± 2 months	CA125> 32 U/mL and NT-proBNP levels> 5,300 pg/mL had independent prognostic value for major adverse events and death.
Vizzardi et al. (65)	2012	Observational, prospective	102	Mild to moderate HF	Cut point: 30 U/mL	43 ± 15 months	In patients with mild to moderate HF receiving optimized treatment, higher plasma CA125 levels is an effective long-term prognostic indicator for predicting cardiovascular events and hospitalization of HF, and may be helpful for better risk stratification.
Miñana et al. (66)	2012	Observational, prospective	293	AHF	Cut point: 35 U/mL	6 months	Elevation of CA125 levels after the first weeks of admission is associated with an increased risk of readmission for AHF.
Zhuang et al. (23)	2014	Meta-analysis	4159 (23 studies)	Acute and chronic HF	Not Reported	Not Reported	Patients with high short-term and long-term mortality showed higher levels of CA125.
Becerra et al. (67)	2017	Observational, retrospective	55	HTx	Cut point: 35 U/mL	48 months	CA125 (>35 U/ml) was the only factor that is independently related to long-term mortality rate.
Kaya et al. (68)	2017	Observational, prospective	267	Acute decompensated HF	Cut point: 48 U/mL,	4 days (The median length of stay)	CA125 is independently associated with prolonged length of stay.
Núñez et al. (69)	2017	Observational, prospective	946	AHF	Cut point: 35 U/mL	2.64 years	The risk is the highest when NT-proBNP (⩾1000 pg/ml) and CA125 (>35 U/ml) rise at the same time.
Li et al. (70)	2018	Meta-analysis	8401 (16 studies)	AHF	Not Reported	Not Reported	High CA125 levels were associated with an increase in the risk of death and HF readmission.
Yoon et al. (71)	2019	Observational, prospective	413	Acute decompensated HF	Cut point: 54.5 U/mL	591 ± 233 days	The ability to predict the mortality of Acute decompensated HF patients is further increased by combining CA125 with NT-proBNP and the identified risk factors.
Núñez et al. (24)	2020	Observational, prospective	2516	Worsening HF	Median: 38.6 U/mL (16-125)	1 year	CA125 is independently associated with a higher risk of clinical outcomes, even exceeding the predefined risk model and clinical substitutes for congestion.
Soler et al. (72)	2020	Observational, prospective	2961	AHF with TR	Median: 58.1 U/mL (26–129)	3.3 ± 3.2 years	Patients with higher CA125 showed a higher long-term all-cause mortality risk, and the effect was better in patients with severe TR.
Núñez et al. (73)	2022	Observational, retrospective	3302	AHF	Median: 57 U/mL (25.3-127)	6 months	In short-term and 6-month follow-up, CA125 identified the subgroups of patients with low risk of hospitalization for death/HF.

CA125, carbohydrate antigen 125; HF, heart failure; AHF, acute heart failure; HTx, heart transplantation; BNP, brain natriuretic peptide; NT-proBNP, N-terminal pro-B-type natriuretic peptide.

### 3.4 CA125: response to treatment

Recent studies indicate that an alteration of CA125 is in line with the evolution of clinical status (**Table 4**). For instance, a study involving 77 patients who underwent heart transplantation revealed that the concentration of CA125 in individuals with a notable improvement in NYHA class after the procedure decreased. Conversely, the level of CA125 in subjects with deteriorating HF increased (5). Similarly, Núñez et al. (76) reported that peritoneal dialysis resulted in a sustained decrease in plasma CA125 levels in patients with advanced congestive HF. This decrease was accompanied by improvements in the patient’s clinical and congestion status, despite the serosal irritation caused by infusion of the osmotic solution into the peritoneum. Further studies have also observed a decrease in CA125 levels with enhanced diuretic therapy (74, 77, 78). Therefore, after effective medication or surgical treatment, serum CA125 decreased with the improvement of clinical symptoms, and vice versa (79).

**Table 4:** Response of CA125 level to treatment.

Author	Year	Study type	N	Population (Treatment methods)	Follow-up	Changes of CA125 levels	Results
Nägele et al. (5)	1999	*In vitro*	71	Chronic HF (HTx)	Without HTx, it was 1.9 ± 0.8 years (0.1 to 3.9 years); after HTx, it was 2.29 ± 1.85 years	CA125 levels after HTx (401 ± 259 U/L vs. 33 ± 22 U/L);worsening of heart failure (42 ± 25 U/L vs. 89 ± 32 U/L)	Neurohormonal levels and hemodynamics were parallel to those of CA125.
D**’**Aloia et al. (7)	2003	Observational, prospective	286	Congestive HF (Medical treatment optimization)	6 ± 3 months	CA125 decreased from 125 ± 98 to 53 ± 61 U/mL (at least 1 NYHA Class reduction)	CA125 was only decreased in patients with improved functional status of NYHA classification, but there were no significant differences in patients with unchanged NYHA classification.
Faggiano et al. (19)	2005	Observational, prospective	30	Chronic HF (High doses of diuretics, intravenous vasodilators and dobutamine)	5–20 days	CA125 decreased from 107 ± 85 to 19 ± 8 U/mL (at least 1 NYHA Class reduction)	When clinical improvement was achieved (at least one NYHA grade was reduced), CA125 was significantly reduced.
Núñez et al. (74)	2012	Observational, prospective	25	Advanced congestive HF with renal dysfunction (CAPD)	6 and 24 weeks	At 6 weeks: 32.2 U/mL (22.1–49.6); at 24 weeks: 28.1 U/mL (18.2–75.6)	After CAPD treatment, the level of CA125 improved with the decline of NYHA functional class at the 6th and 24th weeks.
Núñez et al. (75)	2012	Observational, prospective	293	AHF(Not Reported)	18 (10–32) months	At index hospitalization: 72.6 U/mL (27.8–140.1) At first ambulatory visit: 26.2 U/mL(16.3–63.7)	The normalization of CA125 (< 35 U/mL) is associated with lower risk. Continuous CA125>35U/mL is associated with higher risk.
Miñana et al. (66)	2012	Observational, prospective	293	AHF(Not Reported)	6 months	At index hospitalization(categorical changes): 45 (24-106), 169 (93-255), 68 (17-98) U/mL At first ambulatory visit(categorical changes): 18 (13-23), 66 (49-112), 68 (25-126) U/mL	An elevation of CA125 levels after the first weeks of admission is associated with an increased risk of readmission for AHF.
Núñez et al. (69)	2017	Observational, prospective	946	AHF(Not Reported)	2.64 years	Alive: 66.5 U/mL (27.0–136.0); Deceased: 63.1 U/mL (28.4–141.7)	The risk is the highest when NT-proBNP (⩾1000 pg/ml) and CA125 (>35 U/ml) rise at the same time.
Núñez et al. (76)	2011	Observational, prospective	17	Advanced congestive HF (peritoneal dialysis)	120 days	Baseline:70.86 (46.14–206.7)U/mL; At 45 days after dialysis onset 28.77 (22.09–38.6)U/mL; At 120 days after dialysis onset s 27.47 (16.7–30.05)U/mL	After peritoneal dialysis was started in patients with advanced congestive heart failure, the plasma CA125 continued to decrease, and the clinical and congestion status of patients was improved.
Núñez et al. (77)	2018	Observational, retrospective	25	Refractory congestive HF (acetazolamide to an intensive diuretic regimen)	152 days	CA125 decreased significantly during follow-up	Addition of acetazolamide to an intensive diuretic regimen significantly reduced CA125 in patients with refractory congestive heart failure while improving NYHA class.

CA125, carbohydrate antigen 125; HF, heart failure; AHF, acute heart failure; HTx, heart transplantation; NYHA, New York Heart Association; CAPD, continuous ambulatory peritoneal dialysis; NT-proBNP, N-terminal pro-B-type natriuretic peptide.

### 3.5 CA125 for guiding therapy for heart failure

Diuretics have been shown to alleviate symptoms of pulmonary and systemic venous congestion (1). Paradoxically, studies have shown that the use of higher doses of loop diuretics is associated with a poor prognosis (80, 81), a view shared by Ahmed et al. (82) López-Vilella et al. (78) found that the combination of diuretics required to improve patients with decompensated HF is associated with the CA125 level, site of congestion, renal function, and right ventricular functionality. Compared with patients with pulmonary congestion who used a single diuretic, those with systemic congestion with more diuretic combinations had higher levels of CA125. However, the optimal use boundary of loop diuretics relies heavily on subjective empirical assessment rather than evidence-based guidance. This highlights the need for further research in this area. Notably, CA125 has the potential to be a useful tool in monitoring and guiding diuretic therapy following episodes of AHF (75, 83). A prospective randomized multicenter trial aimed to assess treatment effects based on a series of measurements of serum CA125 levels in HF patients (84). These include adjusting the dose of diuretics based on serum CA125 levels, increasing the number of outpatient visits, administering intravenous iron in patients with iron deficiency, upregulating of statins in those with high serum CA125 levels, and initiating mineralocorticoid antagonists, with the goal of maintaining serum CA125 levels below 35 U/ml. The results suggest that the CA125-guided regimen is superior to the standard regimen and that its effect is primarily driven by a significantly lower rate of readmissions rather than mortality. The study also found that the effect of CA125-guided therapy was time-dependent, with the effect being more pronounced in the first 6 months, after which the Kaplan-Meier curve tended to converge. The study indicates that monitoring serum CA125 levels in patients can aid in detecting and treating congestion early, potentially preventing rehospitalization for HF. However, the serum CA125-guided approaches focus on empirical and sometimes controversial interventions. It is still noteworthy that these therapeutic options regimens for reducing serum CA125 are not specific to relieving congestion. Statins seem to reduce the expression of CA125 by inhibiting the inflammatory response (85, 86), while Cleland et al. (87) found that statins appeared to be ineffective in patients with HF and more severe congestion. From this, it may be inferred that an elevated CA125 was likely to indicate that statins would be ineffective. Likewise, more direct evidence is needed to verify the value of CA125 in optimizing diuretics to relieve congestion.

A significant proportion of patients admitted with AHF exhibit renal dysfunction or experience worsening of renal function during hospitalization. These conditions contribute significantly to prolonged hospital stays and poor prognosis (88, 89). The kidneys play a key role in congestion. Traditionally, reduced cardiac output leads to decreased glomerular filtration and increased tubular reabsorption, resulting in water and sodium retention (69). However, the kidneys themselves may contribute to HF-related congestion before the neurohormonal system intervention (88). Therefore, further consideration should be given to the clinical effectiveness of CA125 levels in adjusting the intensity of diuretic therapy in patients with cardiorenal syndrome. In a randomized study involving 160 patients with AHF and renal dysfunction, a CA125-guided diuretic strategy was found to be more effective than conventional treatment in improving short-term renal function in these patients (90). Other uses for CA125 in cardiovascular medicine.

Given that CA125 is not a specific cardiac biomarker, **Table 5** summarizes the current clinical applications of CA125 in various cardiovascular diseases. Among them, some studies have found that elevated levels of CA125 were associated with an increased risk of new-onset AF in HF patients (101), and the inflammatory response associated with AF may be a key factor in inducing serum CA125 synthesis (91, 102–104). Serum CA125 can independently predict the recurrence of AF after radiofrequency ablation, and patients with serum CA125 levels above 13.75 U/mL had a higher risk of AF recurrence than patients with serum CA125 levels below the critical value (102). A study conducted on 228 patients with severe aortic valve disease revealed that the longitudinal history of CA125 levels can be used to predict adverse clinical outcomes after transcatheter aortic valve implantation (94). When assessing the prognosis of AHF patients with functional TR, the use of CA125 and NT-proBNP yielded different results. That study found that NT-proBNP was significantly associated with mortality in nonsevere TR patients, while elevated levels of CA125 were significantly associated with mortality risk in all patients, with a greater impact on severe TR patients (72). Recent studies have indicated that CA125 may have a positive impact on patients with COPD and PAH who have impaired right heart function (99). Therefore, in addition to aid in diagnosis and treatment, CA125 levels have been found to be correlated with the severity and prognosis of various cardiovascular diseases.

**Table 5:** Research progress of CA125 and other cardiovascular diseases.

Disease	Diagnosis	Echocardiography	Clinical Status	Prognosis	Therapeutic
Atrial fibrillation (91–93)	–	√	√	√	–
Aortic stenosis (58, 94, 95)	–	√	√	√	–
Non-ischemic dilated cardiomyopathy (14)	–	√	√	√	–
Coronary atherosclerotic heart disease (96)	–	√	√	√	–
Hypertrophic cardiomyopathy (9)	–	√	√	–	–
Mitral stenosis (18)	–	√	√	–	–
ST-elevation myocardial infarction (97, 98)	–	√	√	√	–
Chronic obstructive pulmonary disease (13)	–	√	√	√	–
Pulmonary hypertension (99, 100)	–	√	√	√	–

—, Not reported; √, Positive finding.

## 4 Value of CA125 compared to other cardiovascular biomarkers

### 4.1 Added value of CA125 over NPs

Secretion of B-type natriuretic peptides (BNP) is stimulated by increasing atrial and ventricular wall tension, reflecting the degree of congestion. Accordingly, increased plasma concentrations of BNP/NT-proBNP are associated with more severe symptoms of HF and a worse prognosis, and they are also useful markers of therapeutic response (105). CA125 was positively correlated with NT-proBNP, although the correlation were mostly weak (24, 26, 41, 42). However, from a clinical point of view, CA125 was positively correlated with parameters of congestion (serous effusion, peripheral edema, inferior vena cava pressure (106), pulmonary wedge pressure, and severity of TR) (8, 41, 42, 60, 72, 84, 107). That study found that NT-proBNP values were only positively correlated with pleural effusion and IVC diameter, and the correlation was weaker compared to CA125 (69, 77, 83). Another study showed that neither clinical parameters of congestion nor surrogate echocardiographic parameters of right ventricular dysfunction were associated with higher values of NT-proBNP (25). It can be inferred that CA125 is more appropriate to evaluate patients with right ventricular dysfunction. Additionally, studies have reported that CA125 provides better prognostic information than NPs. Notably, as a pathophysiological biomarker different from NT-proBNP, CA125 also complements NT-proBNP in other ways. First, the effectiveness of NT-proBNP in guiding HF therapy occasionally leads to contradictory result, especially in elderly patients, despite their NT-proBNP being apparently improve (26), whereas serum CA125 production was not influenced by age, sex or renal function, which bypasses many of the limitations of NT-proBNP (41). Second, the average half-life of BNP is short, approximately 20 minutes, and its responses to initial therapy vary significantly. CA125, typically having a half-life of more than one week, is a biomarker of delayed response to acute hemodynamic changes. Combining acute hemodynamic information from the initial therapy (provided by BNP) with information on the chronicity of HF (provided by CA125) provides better prognosis and risk stratification (21, 41), which is similar to blood sugar and glycated hemoglobin in diabetic patients. Therefore, for patients with decompensated HF, CA 125 is a reliable surrogate that reflects fluid status over the past few weeks (20, 41, 42).

### 4.2 CA125 and other biomarkers

In addition to the association between proinflammatory cytokines (TNF-α and IL-6) and NPs, CA125 also shows a high affinity for Galectin-1 and Galectin-3. A study (108) reported that Gal-3 was closely associated with a higher risk of long-term death and repeated hospitalization but only in AHF patients with CA125 values greater than 67 U/ml. Furthermore, CA125 levels are also positively correlated with biomarkers that represent filling pressure/congestion, such as BIO-ADM (109). ADM is expressed by various cells including vascular endothelial cells, cardiomyocytes, leukocytes, and vascular smooth muscle cells, and it plays a vital role in maintaining endothelial barrier function. Increased levels of plasma ADM may suggest excessive fluid overload and are significantly related to symptoms and adverse consequences in patients with congestive HF (110–112). Serum BIO-ADM, soluble CD146, and CA125 were categorized together by Boorsma et al. (2) As valuable biomarkers for evaluating tissue congestion, this result provides a new method for evaluating congestion in multiple parameters. However, further research is still needed to compare this marker with the other recognized HF markers.

## 5 Problems and prospects

Undeniably, the following challenges still exist: (1) The pathophysiological role of CA125 upregulation in HF is not clear, and most of the relevant studies remain speculative at the moment. Whether the overexpression of CA125 is a consequence of mechanical stress and inflammatory stimulation caused by congestion or appears as a reason for exacerbating congestion is still being debated. (2) The threshold of CA125 for diagnosing HF has not yet been clarified yet. (3) There was a high level of heterogeneity between studies, and larger prospective studies and more controlled studies are needed to confirm the clinical value of CA125. (4) CA125 does not have organ specificity or disease specificity. In the absence of a diagnosis of HF, elevated CA125 may indicate a wide range of heterogeneous diseases, and thus a comprehensive clinical evaluation is needed.

## 6 Conclusions

The close relationship between CA125 and congestive HF is a surprising discovery, which further distinguishes between intravascular congestion and tissue congestion. To a certain extent, it compensates for the lack of a standardized biomarker for assessing congestion in daily clinical practice. CA125 has the capacity to predict beyond NT-proBNP and has important prognostic, risk stratification, monitor, and treatment guidance functions. Furthermore, its wide availability and low cost facilitate its application in clinical practice.

## Author contributions

RF drafted this manuscript. ZLZ and QKF revised the final version of the manuscript. All authors contributed to the article and approved the submitted version.
